# Proof of Citizenship at the Point of Care: Documentation Requests and Forgone Care Among California Immigrants, 2019–2022

**DOI:** 10.1111/1475-6773.70168

**Published:** 2026-09-15

**Authors:** Nari Yoo

**Affiliations:** ^1^ School of Social Work University of Michigan Ann Arbor Michigan USA

**Keywords:** California health interview survey, chilling effect, forgone care, health care access, immigrant health, limited English proficiency

## Abstract

**Objective:**

To estimate how often immigrant adults are asked for a Social Security number or proof of citizenship to obtain care or enroll in school, who is asked for these documents, and whether being asked relates to health care access.

**Study Setting and Design:**

Cross‐sectional analysis of four pooled waves (2019–2022) of a population‐based, multilingual survey of California adults. The exposure was the documentation request; outcomes were lacking a usual source of care and delaying or forgoing care.

**Data Sources and Analytic Sample:**

California Health Interview Survey public‐use files were used. The documentation item is asked only of foreign‐born respondents, yielding 21,133 immigrant adults (15,313 naturalized, 5820 noncitizen). Survey‐weighted logistic models with jackknife variance reported average marginal effects.

**Principal Findings:**

Overall, 15.7% (95% CI: 14.9–16.5) reported being asked. After adjustment, noncitizens were 5.3 percentage points more likely than naturalized citizens (3.5–7.1) and limited‐English‐proficient adults 5.8 points more likely (3.5–8.1) to be asked. Being asked was associated with delaying or forgoing care (6.1 points; 4.2–8.0) but not with lacking a usual source of care (0.2 points; −1.9 to 2.3).

**Conclusions:**

Documentation requests were common, concentrated among noncitizen and limited‐English‐proficient immigrants, and associated with forgone care. The cross‐sectional design cannot establish causation.

## Summary

- What is known on this topic?
  ○ Immigration enforcement and benefit policies discourage eligible immigrants from using health care, a chilling effect that reaches beyond those any single policy formally covers.
  ○ Most evidence captures immigrants' own avoidance or community‐level enforcement exposure, not the specific encounters that prompt people to withdraw from care.
  ○ Being asked for citizenship documents when seeking care is a plausible but rarely measured trigger of forgone care.
- What this study found?
  ○ About one in six California immigrant adults reported being asked for a Social Security number or proof of citizenship to obtain medical care or enroll in school.
  ○ These requests fell disproportionately on noncitizens and adults with limited English proficiency.
  ○ Being asked for documents was associated with delaying or forgoing needed care, though not with lacking a usual source of care.

## 1 Introduction

A clinic or hospital can ask a patient for documents that have no bearing on the clinical problem at hand: a Social Security number, proof of citizenship or immigration status. For most patients these requests are routine paperwork tied to billing or coverage, and whether they are made depends on registration practice [1]. For an immigrant who worries that contact with an institution could expose their own or a relative's immigration status, the same request at the point of care is a reason to leave without being seen. Immigration enforcement and benefit policy have repeatedly produced this kind of withdrawal from public systems among people who are themselves eligible for care, from Medicaid participation after welfare reform [2] to safety‐net enrollment after the expansion of interior immigration enforcement [3], a chilling effect that reaches well beyond the immigrants any single policy formally covers [4, 5]. The encounter that can prompt such withdrawal has been less examined: being asked, at the moment of seeking care, for documents that signal the institution is checking status.

The 2019 federal public charge rule, in effect during part of the study period, and the broader climate of immigration enforcement generated confusion across immigrant communities about whether using health programs could jeopardize immigration status, and surveys conducted during these years found that a substantial share of adults in immigrant families avoided public benefits or services out of such concerns [6]. The intensity of this climate also varies across states [7]. In pooled California data from 2017 to 2020, Latino immigrants without a green card were 23 percentage points less likely to be insured than citizens, and avoidance of Medicaid persisted even after the rule was reversed [8]. In California, where immigrants make up more than a quarter of residents [9] and where Medi‐Cal eligibility expanded to low‐income adults regardless of immigration status [10, 11], the gap between formal eligibility and actual use affects a large population. That expansion has since narrowed: the 2025–2026 state budget froze new full‐scope Medi‐Cal enrollment for undocumented adults beginning in January 2026 and will add a monthly premium in 2027 [11], so coverage for newly arriving immigrants is contracting.

Prior research has measured the chilling effect in three ways. One body of work measures the immigrant's own avoidance behavior [12]. A second measures community‐level enforcement exposure and links it to downstream care‐seeking [2, 13]. A third, more recent line measures immigrants' direct encounters with enforcement; Young et al. [14], using a California survey of Asian and Latinx immigrants, found that most reported enforcement experiences were associated with delaying care. The documentation request itself has rarely been measured across a population‐based sample of immigrants regardless of enforcement experience. The California Health Interview Survey (CHIS) asks immigrant respondents exactly this, which makes it possible to estimate how common such requests are, who receives them, and how they relate to access. Exposure should not fall evenly across the foreign‐born population. Because these requests arise from eligibility and billing checks, they should follow the characteristics that make eligibility appear uncertain, including noncitizenship, limited English proficiency, youth, low income, and race and ethnicity, and should be less common in safety‐net settings that serve patients regardless of immigration status [15].

We used pooled CHIS data from 2019 to 2022 to ask three questions. How common is being asked for a Social Security number or proof of citizenship to obtain medical care or enroll in school among immigrant adults? Which immigrants are asked for these documents, by citizenship, English proficiency, and interview language? And is being asked associated with two access markers, lacking a usual source of care and delaying or forgoing needed care? The first two questions are descriptive; the third is the primary analysis.

## 2 Methods

### 2.1 Study Design and Population

The California Health Interview Survey is a population‐based survey of California households conducted by the UCLA Center for Health Policy Research using an address‐based, multistage sampling design with interviews in multiple languages [16]. We pooled the public‐use Adult files for survey years 2019 through 2022. The overall adult response rate (the product of screener and extended‐interview rates) was 11.6% for 2019–2020 and 8.6% for 2021–2022. The documentation item is administered only to foreign‐born respondents, so the analytic universe is immigrant adults (naturalized citizens and noncitizens). Of 90,025 pooled adult respondents, 21,133 were immigrant adults who answered the documentation item (15,313 naturalized, 5820 noncitizens), representing approximately 9.5 million California adults per year. Item missingness was negligible; three respondents were missing English proficiency, so adjusted models are estimated on 21,130 respondents without imputation.

### 2.2 Measures

#### 2.2.1 Dependent Variables

Two access outcomes were drawn from the CHIS adult questionnaire and coded consistently across waves. Lacking a usual source of care was coded 1 for respondents with no usual place to go when sick or needing health advice, and 0 otherwise. Delayed or forgone care was coded 1 for those who delayed or did not get a needed medical service in the past 12 months, and 0 otherwise. Both items are administered to all adults.

#### 2.2.2 Independent Variable

The focal exposure was the documentation request, coded 1 for respondents who reported being asked in the past 12 months to provide a Social Security number or show proof of citizenship or legal status when trying to get medical services or enroll in school, and 0 otherwise. Three measures characterized the immigrant respondent. Citizenship status distinguished naturalized citizens (the reference category) from noncitizens. English proficiency distinguished respondents who spoke only English (the reference category), who spoke English well, and who spoke English not well or not at all (limited English proficiency). Interview language distinguished English, Spanish, and an Asian language (Chinese, Korean, Vietnamese, or Tagalog, the other languages in which CHIS is administered).

#### 2.2.3 Covariates

All models adjusted for age in four groups (18–34 [the reference], 35–49, 50–64, and 65 or older), sex (male as reference), educational attainment in four groups (less than high school as reference, high school graduate or some college, vocational or associate degree, and bachelor's degree or higher), family income as a ratio of the federal poverty level (continuous, top‐coded at 6), and survey year (2019 as reference).

### 2.3 Statistical Analysis

All estimates applied the CHIS sampling weights and 80 jackknife replicate weights, with pooled weights divided by four for the four combined waves; variances were estimated by jackknife. For the descriptive questions, we estimated survey‐weighted prevalences of being asked overall and by citizenship, English proficiency, and interview language, testing group differences with design‐based *F* tests. We then estimated the weighted prevalence of each access outcome by whether the respondent was asked, and fit survey‐weighted logistic regressions of being asked, and of each access outcome on being asked, each adjusted for citizenship, English proficiency, and the covariate set. We report adjusted odds ratios with 95% confidence intervals (Tables S2 and S3), average marginal effects, and predicted probabilities. In sensitivity analyses we added race and ethnicity to all three models, re‐estimated them within racial and ethnic groups, and compared the prevalence of being asked by type of usual source of care (Tables S4–S8). Analyses were conducted in Stata/MP 17.

## 3 Results

### 3.1 Sample Characteristics

Table S1 presents the weighted characteristics of the 21,133 immigrant adults by citizenship status. Naturalized citizens made up about three in five weighted immigrant adults and were older, higher‐income, and more likely to speak only English. Noncitizens were more likely to have less than a high school education (38.7% vs. 27.6%), limited English proficiency (35.9% vs. 20.2%), and a Spanish‐language interview (40.7% vs. 18.4%). The two access outcomes diverged: noncitizens were more likely to lack a usual source of care (28.8% vs. 13.8%, *p* < 0.001), while delayed or forgone care was similar (14.7% vs. 14.5%, *p* = 0.75).

### 3.2 Distribution of Documentation Requests

**FIGURE 1:**
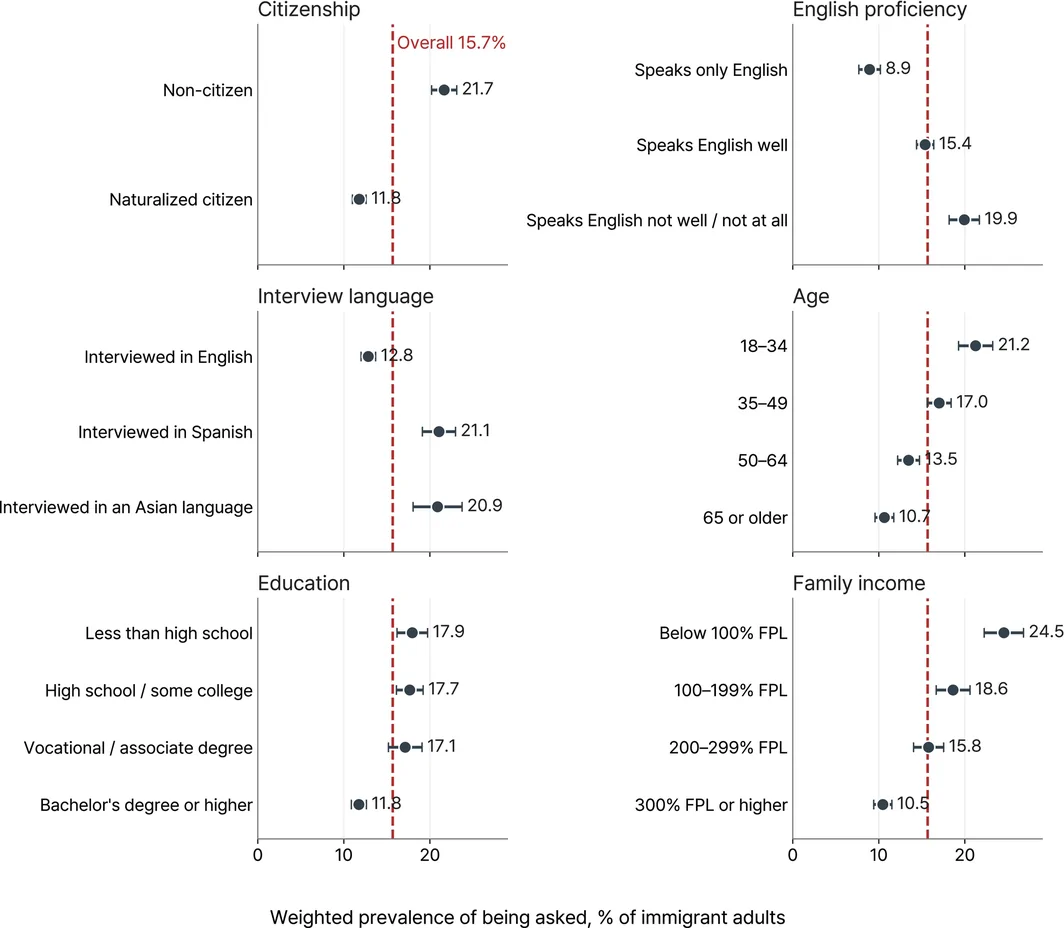
Who is asked for a Social Security number or proof of citizenship to obtain medical care or enroll in school, among California immigrant adults. *Source:* CHIS 2019–2022. Each point is the survey‐weighted prevalence of being asked within a subgroup, with 95% confidence intervals; panels group subgroups by dimension and categories appear in their natural order. The dashed line marks the overall prevalence (15.7%). CI, confidence interval; SSN, Social Security number. Subgroups are defined in Section 2.2.

Across immigrant adults, 15.7% (95% CI: 14.9–16.5) reported being asked for a Social Security number or proof of citizenship to obtain medical care or enroll in school, and prevalence ranged from about 9%–25% across subgroups (Figure 1). Being asked was more common among noncitizens than naturalized citizens (21.7% vs. 11.8%) and among adults with limited English proficiency (19.9%) than English‐only speakers (8.9%), and it declined steadily with age and with income.

These differences persisted after adjustment (Table 1). Noncitizens were more likely to be asked than naturalized citizens (predicted probability 13.3%), by 5.3 percentage points (95% CI: 3.5–7.1). Relative to adults who spoke only English (predicted probability 12.0%), being asked was more likely among those who spoke English well (by 3.4 percentage points, 95% CI: 1.6–5.2) and those who spoke English not well or not at all (by 5.8 percentage points, 95% CI: 3.5–8.1). The probability of being asked declined with age and with income (−2.1 percentage points per federal poverty level unit, 95% CI: −2.6 to −1.7) and was lower in 2020 and 2021 than in 2019. The citizenship and English‐proficiency differences persisted when race and ethnicity were added to the model, and neither race and ethnicity nor the type of usual care setting accounted for them (Tables S4–S8).

**TABLE 1:** Average marginal effects on the probability of being asked for a Social Security number or proof of citizenship to obtain medical care or enroll in school, immigrant adults, CHIS 2019–2022 (*n* = 21,130).

Predictor	AME (pp)	95% CI	Predicted probability (%)
Citizenship (ref: naturalized)			13.3
Noncitizen	+5.3***	3.5, 7.1	18.6
English proficiency (ref: speaks only English)			12.0
Speaks English well	+3.4***	1.6, 5.2	15.4
Speaks English not well or not at all	+5.8***	3.5, 8.1	17.7
Age group (ref: 18–34)			20.0
35–49	−3.4**	−5.7, −1.1	16.6
50–64	−5.9***	−8.2, −3.6	14.1
65 or older	−8.6***	−11.3, −5.9	11.3
Female (ref: male)	−0.1	−1.6, 1.4	15.6
Education (ref: less than high school)			15.3
High school or some college	+0.7	−1.6, 3.0	16.0
Vocational or associate degree	+3.0*	0.5, 5.6	18.3
Bachelor's degree or higher	−0.1	−2.5, 2.3	15.2
Family income (FPL ratio)	−2.1***	−2.6, −1.7	19.8 at 1; 15.1 at 3
Survey year (ref: 2019)			17.4
2020	−2.5*	−4.5, −0.4	14.9
2021	−3.4**	−5.6, −1.3	14.0
2022	−1.1	−3.4, 1.1	16.3

*Note:* AME = average marginal effect (percentage‐point change in the probability of being asked), from a survey‐weighted logistic regression with jackknife variance estimation, averaged over the immigrant subpopulation. Estimates are for foreign‐born adults aged 18 and older in the pooled 2019–2022 California Health Interview Survey public‐use adult files. Predicted probabilities are survey‐weighted predictive margins from the same model, averaged over the immigrant subpopulation; on a category heading row the value shown is the predicted probability of that category's reference group, and for family income the values are at federal poverty level ratios of 1 and 3.

Abbreviations: CI, confidence interval; FPL, federal poverty level; pp, percentage points.

* *p* < 0.05.

** *p* < 0.01.

*** *p* < 0.001.

### 3.3 Documentation Requests and Health Care Access

On both unadjusted measures, access was worse among those asked: 24.4% lacked a usual source of care and 19.7% had delayed or forgone needed care, compared with 18.8% and 13.6% among those not asked. After adjustment (Table 2), the two outcomes separated. The probability of lacking a usual source of care did not differ by whether the respondent had been asked for documents (0.2 percentage points, 95% CI: −1.9 to 2.3; predicted probability 19.6% among those not asked). Those who were asked had a higher probability of delaying or forgoing needed care, by 6.1 percentage points (95% CI: 4.2–8.0), than those who were not (predicted probability 13.6%). Noncitizen status (6.7 percentage points, 95% CI: 5.0–8.5) and limited English proficiency (7.4 percentage points, 95% CI: 4.8–10.0) predicted lacking a usual source of care. Both documentation request estimates were unchanged with race and ethnicity added and within racial and ethnic strata (Tables S5 and S6).

**TABLE 2:** Average marginal effects of being asked for documents on health care access outcomes, immigrant adults, CHIS 2019–2022 (*n* = 21,130).

Predictor	No usual source of care, AME (95% CI)	Delayed/forgone care, AME (95% CI)
Asked for SSN/proof of citizenship (ref: no)	+0.2 (−1.9, 2.3)	+6.1*** (4.2, 8.0)
Citizenship (ref: naturalized)		
Noncitizen	+6.7*** (5.0, 8.5)	−0.2 (−1.7, 1.4)
English proficiency (ref: speaks only English)		
Speaks English well	+1.5 (−0.6, 3.7)	−2.2* (−3.9, −0.5)
Speaks English not well or not at all	+7.4*** (4.8, 10.0)	−3.4** (−5.6, −1.2)
Age group (ref: 18–34)		
35–49	−11.6*** (−14.4, −8.9)	−1.0 (−2.7, 0.7)
50–64	−18.2*** (−21.0, −15.4)	+0.0 (−2.1, 2.1)
65 or older	−21.3*** (−24.6, −18.0)	−3.6** (−6.0, −1.2)
Female (ref: male)	−3.6*** (−5.3, −1.8)	+4.2*** (3.0, 5.5)
Education (ref: less than high school)		
High school or some college	−0.6 (−2.9, 1.6)	+1.6 (−0.3, 3.5)
Vocational or associate degree	−2.3 (−5.5, 0.9)	+5.5*** (2.7, 8.3)
Bachelor's degree or higher	−3.1** (−5.2, −0.9)	+5.8*** (3.6, 7.9)
Family income (FPL ratio)	−1.6*** (−2.1, −1.2)	−0.7*** (−1.1, −0.4)
Survey year (ref: 2019)		
2020	−1.3 (−3.5, 0.9)	+0.4 (−1.6, 2.5)
2021	−0.8 (−2.9, 1.4)	+5.0*** (3.2, 6.8)
2022	+3.9** (1.6, 6.3)	+3.4*** (1.7, 5.1)

*Note:* AME = average marginal effect (percentage points). Each column is a separate survey‐weighted logistic regression with jackknife variance estimation, averaged over the immigrant subpopulation. The reference category for the documentation request is not having been asked. Estimates are for foreign‐born adults aged 18 and older in the pooled 2019–2022 California Health Interview Survey public‐use adult files.

Abbreviations: CI, confidence interval; FPL, federal poverty level; SSN, Social Security number.

* *p* < 0.05.

** *p* < 0.01.

*** *p* < 0.001.

## 4 Discussion

About one in six immigrant adults in California reported being asked for documents to obtain medical care or enroll in school, and the request fell more heavily on noncitizens and on adults with limited English proficiency. Wolstein et al. [12] and the broader chilling‐effect work measure the immigrant's response and infer the institutional encounter behind it; we measure that encounter directly and find it concentrated in the groups for whom immigration concerns are most salient. This aligns with Young et al. [14], who found direct enforcement experiences concentrated among Latinx immigrants and associated with delaying care, and with the worse access among noncitizen Latinos that Ortega et al. [17] documented in earlier CHIS waves; the documentation request is a routine instance of the same status‐marking encounter.

Being asked for documents was associated with an adjusted increase of roughly six percentage points in delaying or forgoing needed care, but not with lacking a usual source of care. The magnitude of the delayed‐care association is consistent with the avoidance‐based estimate in Wolstein et al. [12], though smaller, since being asked is more common and less severe than avoiding programs out of fear. A usual source of care reflects a standing relationship that a single documentation encounter is unlikely to dissolve; in our data that relationship was patterned by citizenship and language, not by the request. Delaying a specific service is more proximal and easier to deter; Young et al. [14] reported the same direction with a different exposure. The usual‐source result is consistent with a structural account: the citizen‐noncitizen gap varies with the state policy environment [18] and erodes under sustained enforcement [13, 19].

After adjustment, adults who spoke English not well or not at all reported less delayed or forgone care than those who spoke only English, even though limited English proficiency predicted lacking a usual source of care and being asked for documents. This is consistent with Chang et al. [20], who found that the higher unadjusted rate of delayed and forgone care among limited‐English‐proficient adults narrowed, and in some specifications reversed, after adjustment for socioeconomic factors. Limited English proficiency was still associated with lacking a usual source of care, as in national analyses [21, 22]. The lower reported delay may reflect differences in how need and unmet need are reported across language groups; it is not a protective effect of limited English proficiency.

Naturalized citizens, who are themselves immigrants, were asked for documents at roughly half the rate of noncitizens, and most immigrant adults in every subgroup were not asked at all. This within‐group spread is relevant to the Latino health paradox, the finding that immigrants, including the undocumented, report better health on several measures than US‐born groups despite worse access [23, 24, 25], because an aggregate estimate averages over members who face very different institutional treatment. If documentation requests fall mainly on the noncitizen and limited‐English‐proficient members of a group, a group‐level access estimate for Latino or Asian immigrants understates the exposure of those members and overstates it for the rest, so paradoxical group averages can coexist with acute barriers for part of the group.

### 4.1 Limitations

The data are cross‐sectional, so the association between being asked for documents and forgoing care does not establish that requests cause forgone care. Reverse pathways are plausible: those most likely to be asked may also be those most likely to defer care, and unobserved legal status may explain both. Public‐use survey data do not separate undocumented immigrants from other noncitizens, and inferring legal status from survey responses is itself error‐prone [26], so we treat noncitizens as a single group and cannot isolate the undocumented, for whom the request is likely most consequential. The survey item is itself ambiguous, recording that a request occurred without distinguishing eligibility verification from deterrent gatekeeping. It also combines requests made when seeking medical care with requests made when enrolling in school, and the public‐use file does not separate the two settings. The exposure and outcomes are self‐reported. Finally, the study covers California, whose Medi‐Cal expansions and large immigrant population make its policy environment distinct; estimates may differ in states with more restrictive coverage.

## 5 Conclusion

Being asked for a Social Security number or proof of citizenship to obtain medical care or enroll in school was common among California immigrant adults, was concentrated among noncitizens and limited‐English‐proficient immigrants, and was associated with delaying or forgoing needed care. Clinicians and health systems can reduce unnecessary documentation requests at intake, clarify that care does not depend on citizenship or a Social Security number, and pair eligibility checks with language assistance and community outreach [27], so that a question meant to establish coverage does not become a reason to leave without care.

## Funding

The author has nothing to report.

## Ethics Statement

This study analyzed de‐identified, publicly available secondary data from the California Health Interview Survey and did not constitute human subjects research under 45 CFR 46; institutional review board approval was therefore not required.

## Conflicts of Interest

The author declares no conflicts of interest.

## Supporting information


**Table S1:** Weighted characteristics of immigrant adults in California by citizenship status, CHIS 2019–2022 (*N* = 21,133).
**Table S2:** Survey‐weighted logistic regression (odds ratios) of being asked for documents, immigrant adults, CHIS 2019–2022 (*n* = 21,130).
**Table S3:** Survey‐weighted logistic regressions (odds ratios) of health care access outcomes on being asked, immigrant adults, CHIS 2019–2022 (*n* = 21,130).
**Table S4:** Being asked for a Social Security number or proof of citizenship, by race and ethnicity: unadjusted prevalence and average marginal effects from the adjusted model with race and ethnicity added, immigrant adults, CHIS 2019–2022 (*n* = 21,130).
**Table S5:** Average marginal effects of being asked for documents on health care access outcomes, with race and ethnicity added, immigrant adults, CHIS 2019–2022 (*n* = 21,130).
**Table S6:** Models re‐estimated within racial and ethnic groups, immigrant adults, CHIS 2019–2022.
**Table S7:** Being asked for a Social Security number or proof of citizenship by the type of place reported as the usual source of care, immigrant adults with a usual source of care, CHIS 2019–2022 (*n* = 18,159).
**Table S8:** Why the racial and ethnic contrast in being asked for a Social Security number or proof of citizenship changes sign after adjustment, immigrant adults, CHIS 2019–2022.

## Data Availability

The California Health Interview Survey public‐use files analyzed in this study are publicly available from the UCLA Center for Health Policy Research (https://healthpolicy.ucla.edu/our‐work/california‐health‐interview‐survey‐chis). Analysis code is available from the author on reasonable request.
