# Supplementary material for: Proof of Citizenship at the Point of Care: Documentation Requests and Forgone Care Among California Immigrants, 2019–2022
Source: Health Serv Res. 2026 Sep 15;61(5):e70168. doi: 10.1111/1475-6773.70168 (PMC13578098; doi:10.1111/1475-6773.70168)
Supplement: Supplementary file 1 [file HESR-61-e70168-s001.docx]

**Supporting Information**

**Supplementary Table S1. Weighted characteristics of immigrant adults in California by citizenship status, CHIS 2019–2022 (N = 21,133).**

| Characteristic | Total (n = 21,133) | Naturalized (n = 15,313) | Non-citizen (n = 5,820) | p |
| --- | --- | --- | --- | --- |
| **Age group** |  |  |  | < .001 |
| 18–34 | 2,674 (20.6) | 1,161 (12.7) | 1,513 (32.7) |  |
| 35–49 | 5,730 (31.5) | 3,488 (26.4) | 2,242 (39.3) |  |
| 50–64 | 7,256 (29.9) | 5,751 (35.2) | 1,505 (21.7) |  |
| 65 or older | 5,473 (18.0) | 4,913 (25.7) | 560 (6.3) |  |
| **Sex** |  |  |  | .023 |
| Male | 9,812 (50.1) | 7,159 (49.1) | 2,653 (51.6) |  |
| Female | 11,321 (49.9) | 8,154 (50.9) | 3,167 (48.4) |  |
| **Education** |  |  |  | < .001 |
| Less than high school | 2,487 (32.0) | 1,411 (27.6) | 1,076 (38.7) |  |
| High school or some college | 4,833 (26.6) | 3,391 (25.3) | 1,442 (28.5) |  |
| Vocational or associate degree | 2,409 (7.0) | 1,927 (8.3) | 482 (4.9) |  |
| Bachelor’s degree or higher | 11,404 (34.5) | 8,584 (38.7) | 2,820 (27.9) |  |
| **English proficiency** |  |  |  | < .001 |
| Speaks only English | 4,702 (14.1) | 3,959 (18.5) | 743 (7.4) |  |
| Speaks English well | 12,635 (59.5) | 9,244 (61.3) | 3,391 (56.8) |  |
| Speaks English not well or not at all | 3,793 (26.4) | 2,107 (20.2) | 1,686 (35.9) |  |
| **Interview language** |  |  |  | < .001 |
| English | 15,714 (65.3) | 12,363 (74.4) | 3,351 (51.4) |  |
| Spanish | 3,306 (27.2) | 1,595 (18.4) | 1,711 (40.7) |  |
| Asian language | 2,113 (7.5) | 1,355 (7.2) | 758 (7.9) |  |
| Family income (FPL ratio), M (SE) | 3.08 (0.02) | 3.38 (0.03) | 2.62 (0.03) | < .001 |
| **Survey year** |  |  |  | .059 |
| 2019 | 4,612 (25.6) | 3,413 (25.4) | 1,199 (25.9) |  |
| 2020 | 4,593 (25.3) | 3,414 (25.4) | 1,179 (25.1) |  |
| 2021 | 6,331 (24.8) | 4,493 (24.1) | 1,838 (25.8) |  |
| 2022 | 5,597 (24.3) | 3,993 (25.1) | 1,604 (23.2) |  |
| Asked for SSN or proof of citizenship (yes) | 2,661 (15.7) | 1,598 (11.8) | 1,063 (21.7) | < .001 |
| No usual source of care (yes) | 3,123 (19.7) | 1,694 (13.8) | 1,429 (28.8) | < .001 |
| Delayed or forgone needed care (yes) | 3,267 (14.6) | 2,341 (14.5) | 926 (14.7) | .752 |

Note. Cells are unweighted counts with survey-weighted column percentages, except family income (survey-weighted mean and SE of the family income to federal poverty level ratio, topcoded at 6). p-values are from design-based F tests comparing naturalized citizens and non-citizens. English proficiency has three missing observations (analytic n = 21,130). SSN = Social Security number; FPL = federal poverty level.

**Supplementary Table S2. Survey-weighted logistic regression (odds ratios) of being asked for documents, immigrant adults, CHIS 2019–2022 (n = 21,130).**

| Predictor | aOR | 95% CI |
| --- | --- | --- |
| **Citizenship (ref: naturalized)** |  |  |
| Non-citizen | 1.51*** | 1.32, 1.73 |
| **English proficiency (ref: speaks only English)** |  |  |
| Speaks English well | 1.34** | 1.14, 1.59 |
| Speaks English not well or not at all | 1.61*** | 1.32, 1.96 |
| **Age group (ref: 18–34)** |  |  |
| 35–49 | 0.79** | 0.68, 0.92 |
| 50–64 | 0.65*** | 0.55, 0.76 |
| 65 or older | 0.50*** | 0.41, 0.62 |
| Female (ref: male) | 0.99 | 0.88, 1.12 |
| **Education (ref: less than high school)** |  |  |
| High school or some college | 1.06 | 0.88, 1.27 |
| Vocational or associate degree | 1.25* | 1.04, 1.51 |
| Bachelor’s degree or higher | 0.99 | 0.82, 1.20 |
| Family income (FPL ratio) | 0.85*** | 0.81, 0.88 |
| **Survey year (ref: 2019)** |  |  |
| 2020 | 0.83* | 0.71, 0.97 |
| 2021 | 0.76** | 0.65, 0.90 |
| 2022 | 0.92 | 0.78, 1.09 |
| Constant | 0.27*** | 0.21, 0.35 |

Note. aOR = adjusted odds ratio; survey-weighted logistic regression with jackknife variance; model F(14, 66) = 25.86, p < .001. FPL = federal poverty level. * p < .05, ** p < .01, *** p < .001.

**Supplementary Table S3. Survey-weighted logistic regressions (odds ratios) of health care access outcomes on being asked, immigrant adults, CHIS 2019–2022 (n = 21,130).**

| Predictor | No usual source of care, aOR (95% CI) | Delayed/forgone care, aOR (95% CI) |
| --- | --- | --- |
| Asked for SSN/proof of citizenship (ref: no) | 1.01 (0.88, 1.17) | 1.57*** (1.38, 1.79) |
| **Citizenship (ref: naturalized)** |  |  |
| Non-citizen | 1.57*** (1.40, 1.76) | 0.99 (0.87, 1.12) |
| **English proficiency (ref: speaks only English)** |  |  |
| Speaks English well | 1.12 (0.95, 1.32) | 0.84* (0.74, 0.96) |
| Speaks English not well or not at all | 1.64*** (1.37, 1.97) | 0.76** (0.64, 0.91) |
| **Age group (ref: 18–34)** |  |  |
| 35–49 | 0.53*** (0.46, 0.61) | 0.92 (0.81, 1.06) |
| 50–64 | 0.33*** (0.28, 0.39) | 1.00 (0.85, 1.18) |
| 65 or older | 0.25*** (0.20, 0.31) | 0.74** (0.60, 0.90) |
| Female (ref: male) | 0.78*** (0.69, 0.88) | 1.42*** (1.27, 1.58) |
| **Education (ref: less than high school)** |  |  |
| High school or some college | 0.96 (0.83, 1.11) | 1.16 (0.97, 1.38) |
| Vocational or associate degree | 0.85 (0.68, 1.06) | 1.57*** (1.26, 1.97) |
| Bachelor’s degree or higher | 0.81** (0.70, 0.94) | 1.61*** (1.33, 1.93) |
| Family income (FPL ratio) | 0.89*** (0.87, 0.92) | 0.94*** (0.91, 0.97) |
| **Survey year (ref: 2019)** |  |  |
| 2020 | 0.91 (0.78, 1.07) | 1.04 (0.86, 1.26) |
| 2021 | 0.95 (0.81, 1.10) | 1.50*** (1.29, 1.74) |
| 2022 | 1.29** (1.10, 1.52) | 1.33*** (1.15, 1.53) |
| Constant | 0.54*** (0.42, 0.70) | 0.13*** (0.10, 0.17) |

Note. aOR = adjusted odds ratio; each column is a separate survey-weighted logistic regression with jackknife variance. Usual-source model F(15, 65) = 45.51, p < .001; delayed-care model F(15, 65) = 14.15, p < .001. SSN = Social Security number; FPL = federal poverty level. * p < .05, ** p < .01, *** p < .001.

**Supplementary Table S4. Being asked for a Social Security number or proof of citizenship, by race and ethnicity: unadjusted prevalence and average marginal effects from the adjusted model with race and ethnicity added, immigrant adults, CHIS 2019–2022 (n = 21,130).**

| Predictor | Prevalence or AME | 95% CI |
| --- | --- | --- |
| **Documentation request, unadjusted prevalence by race and ethnicity** |  |  |
| Latino (n = 7,644) | 17.2 | 16.0, 18.4 |
| White, non-Latino (n = 3,929) | 12.4 | 10.9, 14.0 |
| Black, non-Latino (n = 322) | 16.5 | 10.9, 22.2 |
| Asian, non-Latino (n = 8,895) | 13.9 | 12.9, 14.9 |
| AIAN, other, or two or more races, non-Latino (n = 343) | 22.5 | 16.4, 28.6 |
| **Adjusted model with race and ethnicity added, AME (pp)** |  |  |
| Non-citizen (ref: naturalized) | +5.6*** | 3.8, 7.4 |
| Speaks English well (ref: speaks only English) | +4.8*** | 3.0, 6.5 |
| Speaks English not well or not at all | +7.3*** | 4.9, 9.6 |
| Latino (ref: White, non-Latino) | -4.7*** | -7.3, -2.1 |
| Black, non-Latino | +1.8 | -4.6, 8.3 |
| Asian, non-Latino | -2.5* | -5.0, -0.1 |
| AIAN, other, or two or more races, non-Latino | +8.5* | 2.0, 14.9 |
| Age 35-49 (ref: 18-34) | -3.0** | -5.3, -0.8 |
| Age 50-64 | -5.6*** | -7.9, -3.3 |
| Age 65 or older | -8.7*** | -11.4, -6.0 |
| Female (ref: male) | +0.1 | -1.4, 1.6 |
| High school or some college (ref: less than high school) | +0.2 | -2.2, 2.6 |
| Vocational or associate degree | +2.3 | -0.3, 4.9 |
| Bachelor's degree or higher | -1.4 | -4.0, 1.2 |
| Family income (FPL ratio) | -2.2*** | -2.7, -1.8 |
| 2020 (ref: 2019) | -2.6* | -4.6, -0.5 |
| 2021 | -3.6** | -5.7, -1.4 |
| 2022 | -1.2 | -3.5, 1.0 |

Note. Prevalence rows are survey-weighted percentages of immigrant adults reporting that they were asked in the past 12 months, with jackknife 95% confidence intervals; unweighted subgroup sizes are in parentheses in the first column. Group differences in prevalence are significant (design-based F(3.64, 287.87) = 9.09, p < .001). AME rows are average marginal effects in percentage points from a survey-weighted logistic regression identical to the Table 1 model with race and ethnicity added. Race and ethnicity use the CHIS OMB/Department of Finance recode. AIAN = American Indian or Alaska Native; AME = average marginal effect; CI = confidence interval; FPL = federal poverty level; pp = percentage points. * p < .05, ** p < .01, *** p < .001.

**Supplementary Table S5. Average marginal effects of being asked for documents on health care access outcomes, with race and ethnicity added, immigrant adults, CHIS 2019–2022 (n = 21,130).**

| Predictor | No usual source of care, AME (95% CI) | Delayed/forgone care, AME (95% CI) |
| --- | --- | --- |
| Asked for SSN/proof of citizenship (ref: no) | +0.2 (-1.8, 2.3) | +6.1*** (4.2, 8.0) |
| Non-citizen (ref: naturalized) | +6.5*** (4.7, 8.3) | -0.3 (-1.9, 1.2) |
| Speaks English well (ref: speaks only English) | +1.5 (-0.6, 3.7) | -2.2* (-4.0, -0.3) |
| Speaks English not well or not at all | +7.8*** (5.1, 10.4) | -3.1* (-5.5, -0.6) |
| Latino (ref: White, non-Latino) | +1.0 (-1.7, 3.7) | +1.1 (-0.9, 3.1) |
| Black, non-Latino | +0.1 (-4.7, 4.9) | -1.0 (-5.2, 3.2) |
| Asian, non-Latino | -3.0** (-4.9, -1.0) | -2.4* (-4.2, -0.6) |
| AIAN, other, or two or more races, non-Latino | +3.3 (-4.4, 11.0) | +4.1 (-0.2, 8.4) |
| Age 35-49 (ref: 18-34) | -11.9*** (-14.7, -9.2) | -1.2 (-3.0, 0.5) |
| Age 50-64 | -18.4*** (-21.2, -15.6) | -0.1 (-2.2, 2.0) |
| Age 65 or older | -21.2*** (-24.5, -17.9) | -3.4** (-5.8, -1.0) |
| Female (ref: male) | -3.4*** (-5.2, -1.7) | +4.4*** (3.1, 5.6) |
| High school or some college (ref: less than high school) | -0.1 (-2.4, 2.1) | +1.9* (0.1, 3.7) |
| Vocational or associate degree | -1.5 (-4.7, 1.6) | +6.1*** (3.3, 8.9) |
| Bachelor's degree or higher | -1.4 (-3.9, 1.0) | +7.3*** (5.1, 9.6) |
| Family income (FPL ratio) | -1.5*** (-2.0, -1.1) | -0.7** (-1.0, -0.3) |
| 2020 (ref: 2019) | -1.2 (-3.4, 0.9) | +0.4 (-1.7, 2.5) |
| 2021 | -0.7 (-2.9, 1.5) | +5.1*** (3.3, 6.9) |
| 2022 | +4.0*** (1.7, 6.4) | +3.5*** (1.8, 5.2) |

Note. Each column is a separate survey-weighted logistic regression with jackknife variance, identical to the corresponding Table 2 model with race and ethnicity added. The estimates for being asked are unchanged from Table 2 (no usual source of care +0.2, delayed or forgone care +6.1 percentage points). AIAN = American Indian or Alaska Native; AME = average marginal effect (percentage points); CI = confidence interval; FPL = federal poverty level; SSN = Social Security number. * p < .05, ** p < .01, *** p < .001.

**Supplementary Table S6. Models re-estimated within racial and ethnic groups, immigrant adults, CHIS 2019–2022.**

| Racial or ethnic group | Non-citizen → asked, AME (95% CI) | Speaks English well → asked, AME (95% CI) | Speaks English not well or not at all → asked, AME (95% CI) | Asked → no usual source of care, AME (95% CI) | Asked → delayed/forgone care, AME (95% CI) |
| --- | --- | --- | --- | --- | --- |
| Latino (n = 7,644) | +4.1** (1.6, 6.7) | +7.1*** (3.7, 10.4) | +10.1*** (6.1, 14.1) | +0.9 (-2.8, 4.5) | +6.2*** (3.1, 9.3) |
| White, non-Latino (n = 3,929) | +4.7* (0.7, 8.7) | +1.5 (-1.6, 4.6) | +13.5* (1.8, 25.2) | +0.7 (-3.7, 5.1) | +5.8* (0.8, 10.7) |
| Black, non-Latino (n = 322) | +1.8 (-12.6, 16.1) | +5.1 (-7.6, 17.7) | +55.2* (13.2, 97.2) | +1.6 (-17.1, 20.4) | +11.0 (-5.9, 27.9) |
| Asian, non-Latino (n = 8,892) | +8.2*** (5.1, 11.3) | +3.8** (1.0, 6.6) | +4.2* (0.4, 8.0) | 0.0 (-3.5, 3.5) | +4.1* (0.6, 7.6) |

Note. Each cell is an average marginal effect in percentage points from a survey-weighted logistic regression with jackknife variance, fitted separately within the racial or ethnic group named in the row and adjusted for the full covariate set. Reference categories are naturalized citizen, speaks only English, and not having been asked. Estimates for Black immigrant adults rest on 322 respondents and are imprecise; the group with American Indian or Alaska Native, other, or two or more races is too small to fit separately and is omitted. A model interacting being asked with race and ethnicity rejects homogeneity of the delayed-care effect (design-based F(4, 79) = 3.40, p = .01), driven by the two smallest groups. AME = average marginal effect; CI = confidence interval. * p < .05, ** p < .01, *** p < .001.

**Supplementary Table S7. Being asked for a Social Security number or proof of citizenship by the type of place reported as the usual source of care, immigrant adults with a usual source of care, CHIS 2019–2022 (n = 18,159).**

| Type of usual source of care | Prevalence, % (95% CI) | Adjusted AME, pp (95% CI) |
| --- | --- | --- |
| Doctor's office, Kaiser, or HMO | 12.8 (11.9, 13.7) | Reference |
| Clinic, health center, or hospital clinic | 18.6 (17.2, 20.0) | +1.2 (-0.6, 3.0) |
| Emergency room, other place, or no one place | 15.1 (10.0, 20.3) | -2.3 (-7.0, 2.4) |

Note. Prevalence is the survey-weighted percentage reporting that they were asked in the past 12 months, with jackknife 95% confidence intervals. Adjusted average marginal effects are from a survey-weighted logistic regression of being asked on the type of usual source of care, citizenship, English proficiency, and the full covariate set (n = 18,156). Respondents with no usual source of care are outside this table because the item defining the place is not asked of them. AME = average marginal effect; CI = confidence interval; pp = percentage points.

**Supplementary Table S8. Why the racial and ethnic contrast in being asked for a Social Security number or proof of citizenship changes sign after adjustment, immigrant adults, CHIS 2019–2022.**

|  | Latino | White, non-Latino | Asian, non-Latino |
| --- | --- | --- | --- |
| Average marginal effect on being asked, by model, pp (95% CI) |  |  |  |
| Unadjusted | +4.8 (2.8, 6.7) | Reference | +1.5 (-0.3, 3.3) |
| Plus age, sex, education, family income, survey year | -2.2 (-4.6, 0.1) | Reference | -0.7 (-2.9, 1.5) |
| Plus English proficiency | -4.4 (-7.0, -1.9) | Reference | -2.8 (-5.2, -0.4) |
| Plus citizenship (the Supplementary Table S4 model) | -4.7 (-7.3, -2.1) | Reference | -2.5 (-5.0, -0.1) |
| Fairlie decomposition of the gap against White, non-Latino immigrants |  |  |  |
| Prevalence of being asked, % | 17.2 | 12.4 | 13.9 |
| Gap, pp | +4.8 | NA | +1.5 |
| Gap explained by composition, pp | +10.6 | NA | +4.4 |
| Share of the gap explained, % | 221 | NA | 300 |
| Contribution of limited English proficiency, pp | +5.3 | NA | +3.1 |
| Contribution of family income, pp | +4.2 | NA | +0.9 |
| Contribution of non-citizenship, pp | +1.3 | NA | +0.4 |
| Contribution of age 65 or older, pp | +1.2 | NA | +0.6 |
| Group composition, immigrant adults |  |  |  |
| Non-citizen, % | 47.4 | 28.4 | 31.4 |
| Speaks English not well or not at all, % | 35.2 | 4.8 | 22.2 |
| Speaks only English, % | 4.2 | 45.7 | 15.0 |
| Unweighted n with limited English proficiency | 2,032 | 102 | 1,650 |

Note. Average marginal effects are from survey-weighted logistic regressions with jackknife variance estimation, averaged over the immigrant subpopulation (n = 21,130). The middle panel reports a Fairlie nonlinear decomposition, which suits a binary outcome, estimated with the survey weights and 100 randomization replications; a positive value means the characteristic pushes the comparison group above White non-Latino immigrants. Composition more than fully accounts for both raw gaps, with limited English proficiency and family income the largest contributors. English proficiency and citizenship lie on the pathway from race and ethnicity to a documentation request, so the adjusted contrasts estimate a direct effect net of those pathways and answer a different question from the unadjusted differences. Only 102 of 3,929 White non-Latino immigrant adults report limited English proficiency. NA = not applicable; pp = percentage points; CI = confidence interval.
